# How to Investigate the Effects of Groups on Changes in Longitudinal Patient-Reported Outcomes and Response Shift Using Rasch Models

**DOI:** 10.3389/fpsyg.2020.613482

**Published:** 2020-12-23

**Authors:** Karima Hammas, Véronique Sébille, Priscilla Brisson, Jean-Benoit Hardouin, Myriam Blanchin

**Affiliations:** ^1^U1246 SPHERE “methodS in Patient centered outcomes and HEalth ResEarch”, Université de Nantes, Université de Tours, INSERM, Nantes, France; ^2^Methodology and Biostatistics Unit, CHU of Nantes, Nantes, France

**Keywords:** patient-reported outcomes, longitudinal data, Rasch measurement theory, response shift, measurement invariance

## Abstract

In order to investigate patients’ experience of healthcare, repeated assessments of patient-reported outcomes (PRO) are increasingly performed in observational studies and clinical trials. Changes in PRO can however be difficult to interpret in longitudinal settings as patients’ perception of the concept being measured may change over time, leading to response shift (longitudinal measurement non-invariance) and possibly to erroneous interpretation of the observed changes in PRO. Several statistical methods for response shift analysis have been proposed, but they usually assume that response shift occurs in the same way in all individuals within the sample regardless of their characteristics. Many studies aim at comparing the longitudinal change of PRO into two groups of patients (treatment arm, different pathologies, …). The group variable could have an effect on PRO change but also on response shift effect and the perception of the questionnaire at baseline. In this paper, we propose to enhance the ROSALI algorithm based on Rasch Measurement Theory for the analysis of longitudinal PRO data to simultaneously investigate the effects of group on item functioning at the first measurement occasion, on response shift and on changes in PRO over time. ROSALI is subsequently applied to a longitudinal dataset on change in emotional functioning in patients with breast cancer or melanoma during the year following diagnosis. The use of ROSALI provides new insights in the analysis of longitudinal PRO data.

## Introduction

Patient-reported outcomes (PRO) are increasingly used to assess patients’ perception and experience of healthcare with the investigation of health-related quality of life (HRQoL), fatigue, anxiety, and coping (Snyder et al., 2013; Vodicka et al., 2015; LeBlanc and Abernethy, 2017; Lashbrook et al., 2018). These unobserved constructs are often referred to as “latent variables” or “latent traits.” For instance, HRQoL cannot be directly observed nor measured but it can be indirectly investigated using questionnaires in which different items are usually grouped in several domains such as physical, psychological, emotional or social functioning.

Patient-reported outcome measures (PROM) data are directly reported by the patients without interpretation of their responses by a clinician or anyone else and pertains to the patient’s perceived health, HRQoL or emotional functioning. PROM data can inform healthcare decisions by providing the perspectives, interests and values of patients to the healthcare providers on the effectiveness of interventions and health management (Basch, 2017). Still, researchers, policy-makers and physicians are often faced with conceptual, methodological, interpretational, and practical issues regarding the measurement of PRO and interpretation of PROM data to better understand how patients feel, live and adapt to their disease experience and the impact of patients’ perception on prognosis.

Patient-reported outcome measures data are indeed difficult to analyze and interpret for many reasons, especially in longitudinal settings. Indeed, the cognitive processes involved in completing questionnaires are complex (Rapkin et al., 2017) and data are often missing not at random (e.g., patients might be too tired to fill in a fatigue questionnaire). The growing interest in the analysis and interpretation of longitudinal PROM data logically appears in the context of chronic diseases where patients have to regularly adapt to their illness. As a consequence, patients might give different answers to questionnaires over time, not only because their health has changed, but also because their perception of what health or HRQoL means to them has changed. This phenomenon is referred to as response shift (RS) (Sprangers and Schwartz, 1999). RS may result from changes in the patient’s internal standards of measurements (recalibration), changes in his/her values (reprioritization), and changes in his/her definition of the latent trait (reconceptualization). In case of RS, it has been argued on the one hand that it might be impossible to disentangle, without appropriate methodology, “true” perceived HRQoL changes from RS effects (viewed as longitudinal measurement non-invariance), which is problematic for the interpretation of change and of possible intervention effects. On the other hand, the therapeutic importance of patients’ adaptation and of a better understanding of how patients adjust (or not) to their illness and life circumstances can also be highlighted. In the theoretical model of RS and quality of life (Sprangers and Schwartz, 1999), it is assumed that RS may be triggered by mechanisms (e.g., coping, social comparison) in response to a catalyst (a salient health event). In this model, RS is considered as an important mediator of adjustment to illness for patients confronted with a life-threatening or chronic disease. An additional value of including RS in the evaluation of psychosocial or medical intervention studies could be to capture the full treatment impact as treatments that induce RS may be more tolerable (Sprangers and Schwartz, 1999). In fact, patients might shift their internal standards of side effects measurement to better tolerate the side effects of treatments. It has also been reported that when cancer-related fatigue increases substantially as a consequence of treatment, a change in internal standard of measurement, by considering the new level of fatigue as normal, is a desirable adaptation for the patients to cope with the consequences of cancer and its treatment (Visser et al., 2000). RS, closely linked to adaptation, may be one of the goals of therapy in helping patients to cope with their illness and to live with it. In rehabilitation, RS might have a role to improve HRQoL when no improvement can be expected from treatment (Howard et al., 2011). Hence, rehabilitation professionals have the potential to use appropriate care and patient education to trigger the RS process that may result in a change of scale of reference to appropriate goals and improved perceived quality of life. Whatever the adopted viewpoint, we can see that it is important to be able to assess changes experienced by patients, taking into account RS if appropriate, and to detect and quantify RS in a reliable and unbiased manner.

Several statistical methods have been proposed for RS analysis at the domain level such as Structural Equation Modeling (SEM) (Oort, 2005), Classification and Regression Trees (CART) (Lix et al., 2013), and mixed-effects regression models (Lowy and Bernhard, 2004). At the item level, methods based on longitudinal SEM (Oort’s procedure) (Verdam et al., 2016), Item Response Theory (IRT) models (RespOnse Shift ALgorithm at Item-level, ROSALI) (Guilleux et al., 2015) and models from Rasch Measurement Theory (ROSALI) (Blanchin et al., 2020), have been proposed for the detection, interpretation and adjustment for RS in health science.

Focusing on methods coming from Rasch Measurement Theory (RMT) (Andrich, 2004) using random effects Rasch models could be an interesting approach to provide insights into item-level analyses and interpretation of RS. The performance of ROSALI based on IRT or RMT has been assessed in a simulation study (Blanchin et al., 2020) and compared to Oort’s procedure used at item-level. The performance of ROSALI based on RMT outperformed the two other methods in terms of recalibration RS detection, and identification of items affected by recalibration RS.

However, ROSALI as well as most of the methods for RS detection assumes homogeneous RS within the sample (i.e., the majority of patients have adapted to their disease in the same way), regardless of individual clinical or psychological characteristics or study design, e.g., clinical trial with treatment groups, which does not seem realistic (Salmon et al., 2017). Investigating the impact of covariates both on RS and on latent trait change can provide valuable information, either in observational studies or in controlled trials to identify patients’ characteristics on which action can be taken to favor adaptation to illness (assumed to trigger RS) and enhance well-being (as an example of a construct). Moreover, exploring whether RS occurrence and magnitude is similar or not according to covariates of interest (e.g., gender, treatment, cancer site…) can give more insight into patients’ adaptation and adjustment to illness among subgroups.

To overcome the restrictive assumption of homogeneous RS within a sample, especially in studies designed for group comparison, one can incorporate the group covariate in the models to examine whether group membership is associated with RS and PRO change. However, an additional issue arises as patients might have a different perception of the measured PRO depending on their group membership at a specific time point, a phenomenon known as differential item functioning (DIF). Integrating covariates in cross-sectional IRT or RMT models used as latent regression models is quite common to investigate DIF between known (e.g., gender, age…) or unknown groups (latent classes) (Golia, 2015; Cho et al., 2016; Davis et al., 2020; Kleppang et al., 2020) for detecting so-called “biased” items and assessing their impact on the latent trait parameters’ estimates. Although longitudinal IRT or RMT models could also be used in the same way to investigate RS or lack of longitudinal invariance depending on covariates, longitudinal invariance is rarely investigated with IRT or RMT models and covariates are seldom included in such models. One example was found in oral health using IRT (Graded Response Model) (Yau et al., 2018) with the purpose of identifying the covariates associated with oral HRQoL and its change over time adjusting for the lack of longitudinal invariance in some items, but covariates’ effects on RS was not examined.

Before investigating RS with ROSALI in studies designed for group comparison, it is critical to assess the measurement invariance between groups at the first time of measurement i.e., the group effect on item functioning, and to account for non-invariance between groups, if detected, in the subsequent RS detection. Plus, assessing whether RS occurs in the same way in both groups or differentially in each group has to be considered.

Our aim is to present a new version of the ROSALI algorithm based on RMT and how it can incorporate a group covariate to investigate its effect simultaneously on item functioning at the first time of measurement, response shift occurrence (recalibration), and on latent trait change over time. We first detail the two parts of the ROSALI algorithm with the assessment of measurement invariance between groups at the first time of measurement and the detection of RS over two times of measurement, and the statistical models on which it relies. Subsequently, we present an illustrative application of the ROSALI algorithm on a longitudinal study which aim was to compare HRQoL change over time between melanoma and breast cancer patients. We finally discuss perspectives that arise from this work.

## Rosali Algorithm for Detection and Estimation of Group Effects on Item Functioning, Recalibration Response Shift and Latent Trait Change

In its current version, ROSALI is a four-step algorithm for recalibration RS detection at item-level between two time points. In the first step, a flexible model is fitted assuming response shift on all items. In the second step, a constrained model is fitted assuming no RS at all, i.e., longitudinal measurement invariance on all items. These two models are compared using a likelihood ratio test to test for the overall absence of RS. If the test is not significant, no RS is assumed and step 3 is left apart to perform step 4. If the test is significant, RS is suspected and step 3 is performed to go deeper into RS detection. Indeed, in the third step, constraints related to RS are relaxed one by one to identify the items affected by RS and model 2 is improved iteratively by constant update to obtain a model accounting for all identified RS. Finally, in the fourth step, longitudinal PRO change over time is assessed, adjusted for identified RS if appropriate.

To investigate the effect of group on item functioning at the first time of measurement, response shift occurrence (recalibration) and on latent trait change over time, the measurement invariance between groups at the first time of measurement has to be assessed prior to RS detection. The RS detection in step 3 of ROSALI algorithm has to consider different new scenarios. When an item is affected by RS: i/RS can occur in the same way in both groups, ii/RS can occur in one group only, or iii/RS can occur differently in each group. The entirety of the iterative step 3 has to be reconsidered to account for this new framework alongside integrating in all steps the assessment of group effect on longitudinal latent trait change.

**FIGURE 1:**
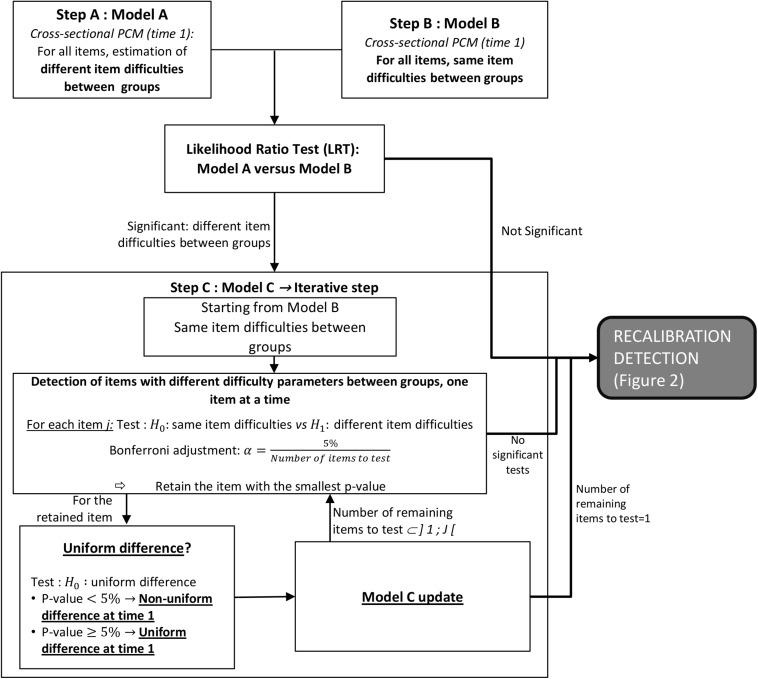
Steps A–C of the ROSALI algorithm: detection of different item difficulty parameters between groups. The cross-sectional PCM at time 1 is detailed in Eq. 2. Uniform difference refers to same differences in item difficulty parameters for all response categories. LRT, likelihood ratio test; PCM, partial credit model.

**FIGURE 2:**
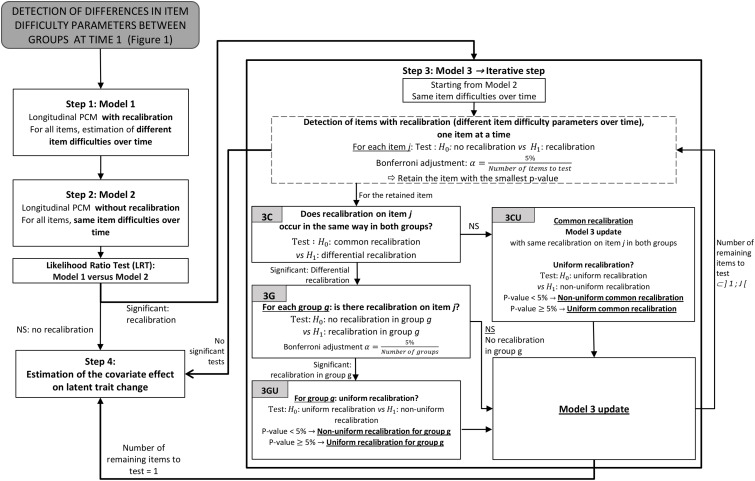
Steps 1–4 of the ROSALI algorithm: recalibration detection and estimation of a covariate effect on latent trait. The longitudinal PCM is detailed in Eq. 3. Uniform recalibration refers to same differences in item difficulty parameters for all response categories. LRT, likelihood ratio test; NS, not significant; PCM, partial credit model, RS, response shift.

The new version of ROSALI algorithm is divided into two parts: the first one (steps A–C, Figure 1) aims at identifying differences in item difficulty parameters between groups at the first measurement occasion, and the second one (steps 1–4, Figure 2) allows for detection of differential recalibration response shift between groups and over time and for estimation of covariate effects on latent trait change between two measurement occasions, time 1 and time 2. The application of ROSALI algorithm presumes that the Partial Credit Model (PCM), presented thereafter, fits adequately the data. The procedure has been automated using Stata (*Stata Statistical Software: Release 15*. College Station, TX: StataCorp LLC) and the Stata module is stored at Boston College’s Statistical Software Components archive (Blanchin and Brisson, 2020).

### Models to Investigate Differences in Item Difficulty Parameters Between Groups at Time 1

The first part of the algorithm consists in three steps (step A–C, Figure 1).

Let *N* be the number of patients answering to a questionnaire including *J* items. *X*_*ij*_ is the response of patient *i* (*i* = 1, …, *N*) to item *j* (*j* = 1, …, *J*).

A cross-sectional PCM is used to model patients’ responses to the items (Masters, 1982; Fischer and Ponocny, 1994). The PCM is a model from the Rasch measurement theory adapted to polytomous items. Each polytomous item *j* is composed of (*m*_*j*_ + 1) response categories numbered from 0 to *m*_*j*_ and therefore has *m*_*j*_ item difficulty parameters. The probability for a patient *i* to respond the response category *x* (*x* = 0, 1, …, *m*_*j*_) of item *j* is a function of:

(i) the latent trait Θ, a random variable assumed to be normally distributed with a mean μ and a variance σ^2^ (θ_*i*_ is a realization of Θ for patient *i*). The latent trait represents the PRO of interest.

(ii) the item difficulty parameters δ_*jp*_ associated with each response category *p* > 0 of item *j* (1 *≤ p ≤ *m*_*j*_*). The item difficulty parameters correspond to the characteristics of the questionnaire.

The cross-sectional PCM is written as follows:

(1) $$P\left(X_{i\,j}=x|\theta_{i},\delta_{j\,1},\ldots ,\delta_{j\,m_{j}}\right)=\frac{\exp\left(x\,\theta_{i}-\sum_{p=1}^{x}\delta_{j\,p}\right)}{\sum_{l=0}^{m_{j}}\exp\left(l\,\theta_{i}-\sum_{p=1}^{l}\delta_{j\,p}\right)}$$

The model parameters are estimated using marginal maximum likelihood (MML). The identifiability constraint μ = 0 is used.

A cross-sectional PCM integrating a group effect on the latent trait and different item difficulty parameters between groups is written as follows:

(2) $$P\left(X_{i\,j}^{\left(1\right)}=x|\theta_{i}^{\left(1\right)},\beta ,g_{i},\delta_{j\,1\,g}^{\left(1\right)},\ldots ,\delta_{j\,m_{j}\,g}^{\left(1\right)}\right)=\frac{\exp\left(x\,\left(\beta \,g_{i}+\theta_{i}^{\left(1\right)}\right)-\sum_{p=1}^{x}\delta_{j\,p\,g}^{\left(1\right)}\right)}{\sum_{l=0}^{m_{j}}\exp\left(l\,\left(\beta \,g_{i}+\theta_{i}^{\left(1\right)}\right)-\sum_{p=1}^{l}\delta_{j\,p\,g}^{\left(1\right)}\right)}\,$$

The group effect on the latent trait is estimated in a latent regression. The latent trait is now divided in two parts: β*g*_*i*_ corresponds to the component of the latent trait explained by the group membership with β the regression coefficient associated to the group of individual *i*, *g*_*i*_, and $\theta_{i}^{\left(t\right)}$ is the residual component corresponding to the latent trait level of individual i at time 1. We have: $\Theta ∼\text{N}\,\left(\mu^{\left(1\right)};\sigma_{1}^{2}\right)$ with μ^(1)^ the mean of the latent trait at time 1 and $\sigma_{1}^{2}$ its variance. The identifiability constraint μ^(1)^ = 0 is used. β is the group effect on the latent trait; *g*_*i*_ = 0 if patient *i* is in group 0, *g*_*i*_ = 1 if patient *i* is in group 1. Therefore, for group 0: $\mu_{0}^{\left(1\right)}=\mu^{\left(1\right)}$ = 0 and for group 1: $\mu_{1}^{\left(1\right)}=\mu^{\left(1\right)}+\beta$ = β. The variances of the latent trait are equal between groups $\sigma_{10}^{2}=\sigma_{11}^{2}=\sigma_{1}^{2}$.

As item functioning may be different depending on group membership, $\delta_{j\,p\,g}^{\left(1\right)}$, the difficulty parameter of the response category *p* of item *j* for group *g* at time 1, can be different across groups.

### Steps A to C: Algorithm for the Detection of Differences in Item Difficulty Parameters Between Groups at Time 1

#### Step A: Fitting a Flexible Cross-Sectional PCM (Model A)

The first detection step consists in establishing a flexible model using a cross-sectional PCM (Eq. 2) at the first measurement occasion estimating different item difficulty parameters for all items between the two groups defined by the covariate *g* (*g* = 0, 1), (Figure 1, Model A). For this model, all item difficulty parameters are different across groups: $\delta_{j\,p\,0}^{\left(1\right)}\neq \delta_{j\,p\,1}^{\left(1\right)}\,\forall j,p$. For identifiability, the additional constraint β = 0 is added (the group effect on the latent trait is not estimated).

#### Step B: No Difference Cross-Sectional PCM and Overall Assessment of Differences in Item Difficulty Parameters Between Groups (Model B)

The second step consists in estimating a constrained model assuming equal item difficulty parameters between groups (Figure 1, Model B). Hence, the constraint $\delta_{j\,p\,0}^{\left(1\right)}=\delta_{j\,p\,1}^{\left(1\right)}\,\forall j,p$ is added and the group effect can be estimated (β≠0).

We test for overall differences in item difficulty parameters between groups to compare the constrained model (Model B) with the flexible model (Model A). A likelihood ratio test (LRT) is performed as Model B is considered equivalent to a model nested in Model A (Gunn, 2020; Sébille et al., 2020). In case of a significant test at 5% level of significance, we proceed to the next step (step C) to detect which items are evidenced as having significant differences in difficulty parameters between groups at time 1. If the test is not significant, we assume that the item difficulty parameters are equivalent between groups and we directly proceed to the second part of the algorithm, the recalibration detection steps starting with step 1 (Figure 2).

#### Step C: Detection of Items With Different Difficulty Parameters Between Groups

Step C is an iterative step starting from the constrained model (Model B) (Figure 1, Model C). *J* different models are estimated. For each model, the equality constraint of item difficulty parameters is relaxed for one item *j* (*j* = 1,…, *J*). The hypothesis of equality of difficulty parameters for this unconstrained item $H_{0}:\forall p,\delta_{j\,p\,0}^{\left(1\right)}=\delta_{j\,p\,1}^{\left(1\right)}$ is then tested with a Wald test. Multiple testing in this iterative step C is taken into account by applying a Bonferroni correction ($\alpha =\frac{5\%}{\mathrm{Number}\,\mathrm{of}\,\mathrm{items}\,\mathrm{to}\,\mathrm{be}\,\mathrm{tested}}$). In case of no significant tests, we subsequently proceed to the last step (step 4). Otherwise, we retain the model with the item *j* having the most significant test (smallest *p* value) among the models for which tests of equality in item difficulty parameters between groups are significant. For this item, we then determine whether the difference in item difficulty parameters between groups is the same for all response categories in which case it will correspond to so-called uniform difference. A Wald test at 5% level of significance is used to test for uniform difference in item *j* difficulty parameters: $H_{0}:\forall p,\delta_{j\,p\,1}^{\left(1\right)}-\delta_{j\,p\,0}^{\left(1\right)}=\Delta_{j}$. If the test is significant, non-uniform difference is evidenced. Uniform difference is assumed otherwise and the associated model is then constrained as follows: $\forall p,\delta_{j\,p\,1}^{\left(1\right)}-\delta_{j\,p\,0}^{\left(1\right)}=\Delta_{j}$. The model is updated to take into account (non-)uniform differences in item difficulty parameters between groups on item *j* if appropriate, and step C is repeated on this updated model to identify differences on the remaining items. The iterative process is stopped when no more differences are detected or when differences have been detected on *J*−1 items among the *J* items, and we subsequently proceed to the first step of recalibration detection.

### Recalibration Detection

This part of the algorithm intends to detect uniform or non-uniform recalibration, i.e., whether difference in item difficulty parameters across time is the same for all response categories or not, respectively. It also aims to identify whether the difference in item difficulty parameters between the first and the second measurement occasion differs between the groups defined by the covariate g. It consists in four steps (step 1 to step 4) that are based on longitudinal PCMs (Figure 2). All longitudinal models derive from the last updated model C to take account of the differences in item difficulty parameters between groups detected in the first part.

The probability for a patient *i* to respond the response category *x* of item *j* at time *t* is modeled as follows:

(3) $$P\left(X_{i\,j}^{\left(t\right)}=x|\theta_{i}^{\left(t\right)},\beta ,g_{i},\beta_{\text{inter}},t_{2},\delta_{j\,1\,g}^{\left(t\right)},\ldots ,\delta_{j\,m_{j}\,g}^{\left(t\right)}\right)=\frac{\exp\left(x\,\left(\beta \times g_{i}+\beta_{\text{inter}}\times t_{2}\times g_{i}+\theta_{i}^{\left(t\right)}\right)-\sum_{p=1}^{x}\delta_{j\,p\,g}^{\left(t\right)}\right)}{\sum_{l=0}^{m_{j}}\exp\left(l\,\left(\beta \times g_{i}+\beta_{\text{inter}}\times t_{2}\times g_{i}+\theta_{i}^{\left(t\right)}\right)-\sum_{p=1}^{l}\delta_{j\,p\,g}^{\left(t\right)}\right)}\,$$

With $\left[\begin{array}{l} \Theta^{\left(1\right)}\\ \Theta^{\left(2\right)} \end{array}\right]\sim \text{N}\left(\left[\begin{array}{l} {\text{ μ}}^{\left(1\right)}\\ {\text{ μ}}^{\left(2\right)} \end{array}\right];\;\sum \right)$

μ^(1)^and μ^(2)^ are the means of the latent trait at times *t* = 1 and *t* = 2, respectively, and $\sum =\left[\begin{array}{c} \sigma_{1}^{2}\;\sigma_{1,2}\\ \sigma_{1,2}\;\sigma_{2}^{2} \end{array}\right]$ is the variance-covariance matrix ($\theta_{i}^{\left(t\right)}$ is a realization of Θ for patient *i* at time *t*).

We have:

• *t*_2_ is the time indicator for *t* = 2: t_2_ = 0 if *t* = 1, *t*_2_ = 1 if *t* = 2

• *g*_*i*_ is the covariate indicator: *g*_*i*_ = 0 if patient *i* is in group 0, *g*_*i*_ = 1 if patient *i* is in group 1

•$\delta_{j\,p\,g}^{\left(t\right)}$ is the difficulty parameter of response category *p* of item *j* for group *g* at time *t.* Similarly to the previous version of ROSALI based on RMT, $\delta_{j\,p\,g}^{\left(t\right)}$ are estimated at each time.

•β is the group effect parameter

•β_inter_ is the interaction parameter between group and time

• The identifiability constraint μ^(1)^ = 0 is used

• For group 0: $\mu_{0}^{\left(1\right)}=\mu^{\left(1\right)}$ and $\mu_{0}^{\left(2\right)}=\mu^{\left(2\right)}$

• For group 1: $\mu_{1}^{\left(1\right)}=\mu^{\left(1\right)}+\beta$ and $\mu_{1}^{\left(2\right)}=\mu^{\left(2\right)}+\beta +\beta_{\text{inter}}$

• The variances of the latent trait at time *t* for group *g*,$\sigma_{t\,g}^{2}$, are equal between groups and freely estimated over time: $\sigma_{10}^{2}$ = $\sigma_{11}^{2}$ and $\sigma_{20}^{2}$ = $\sigma_{21}^{2}$

#### Step 1: Establishing a Recalibration Model (Model 1)

For this model (Figure 2, Model 1), recalibration on all items is considered, that is all item difficulty parameters $\delta_{j\,p\,g}^{\left(2\right)}$ are freely estimated. Hence, no equality constraints are imposed on item difficulty parameters $\delta_{j\,p\,g}^{\left(t\right)}$ over time and on item difficulty parameters at time 2 $\delta_{j\,p\,g}^{\left(2\right)}$ between groups.

Constraints from the first part of ROSALI taking into account the results found in the previous step C (Figure 1) are applied:

•$\delta_{j\,p\,0}^{\left(1\right)}=\delta_{j\,p\,1}^{\left(1\right)}$ if no difference in item difficulty parameters between groups at time 1 was detected on item *j* in step C

•$\forall p,\delta_{j\,p\,1}^{\left(1\right)}-\delta_{j\,p\,0}^{\left(1\right)}=\Delta_{j}$ if a uniform difference in item difficulty parameters between groups at time 1 was detected on item *j* in step C.

Constraints for identifiability are:

• Nullity of the mean of the latent trait for group 0 at time 1: $\mu_{0}^{\left(1\right)}=0$

• Equality of the means of the latent trait over time : $\mu_{0}^{\left(2\right)}=\mu_{0}^{\left(1\right)}=0$ and $\mu_{1}^{\left(2\right)}=\mu_{1}^{\left(1\right)}$

$•\beta_{\text{inter}}=0$

The group effect β is estimated whereas the time effect μ^(2)^−μ^(1)^ and the interaction β_inter_ are constrained to 0 for the model based on Eq. 3 to be identifiable.

#### Step 2: No Recalibration Model (Model 2) and Overall Evaluation of Recalibration

This step consists in estimating a constrained model (Figure 2, Model 2) assuming no recalibration for all items. The model is constrained as follows:

• No recalibration constraints: $\delta_{j\,p\,g}^{\left(1\right)}=\delta_{j\,p\,g}^{\left(2\right)}\,\forall j,p,g$ (no change in item difficulty parameters over time)

• Identifiability constraint: nullity of the mean of the latent trait for group 0 at time 1: $\mu_{0}^{\left(1\right)}=0$

• Constraints from step C:

∘$\delta_{j\,p\,0}^{\left(1\right)}=\delta_{j\,p\,1}^{\left(1\right)}$ if no difference in item difficulty parameters between groups at time 1 was detected on item *j* in step C

∘$\forall p,\delta_{j\,p\,1}^{\left(1\right)}-\delta_{j\,p\,0}^{\left(1\right)}=\Delta_{j}$ if a uniform difference in item difficulty parameters between groups at time 1 was detected on item *j* in step C.

In Model 2, the group, time, and interaction effects are estimated. A likelihood ratio test *(LRT)* is used to compare the constrained model (Model 2, no recalibration) with the flexible model (Model 1, recalibration). In case of a significant test at 5% level of significance, we proceed to the next step (step 3) to identify items affected by recalibration. If the test is not significant, Model 2 is retained and all item difficulty parameters are assumed to be the same over time. In this case, we proceed to step 4 keeping the equality constraints on item difficulty parameters over time $\delta_{j\,p\,g}^{\left(2\right)}=\delta_{j\,p\,g}^{\left(1\right)}\,\forall j,p,g$.

#### Step 3: Detection of Items With Recalibration (Model 3)

Step 3 starts from the constrained model (Model 2) and *J* different models are estimated. For each model, equality constraint of item difficulty parameters is relaxed for one item *j* (*j* = 1,…, *J*). For each model, the hypothesis of equality of the difficulty parameters for this unconstrained item *j* is then tested with a Wald test ($H_{0}:\forall p,g\,\delta_{j\,p\,g}^{\left(1\right)}=\delta_{j\,p\,g}^{\left(2\right)}$). Multiple testing in this iterative step is taken into account by applying a Bonferroni correction ($\alpha =\frac{5\%}{\mathrm{Number}\,\mathrm{of}\,\mathrm{items}\,\mathrm{to}\,\mathrm{be}\,\mathrm{tested}}$). In case of no significant tests, we subsequently proceed to the last step (step 4). Otherwise, we retain the item *j* associated with the most significant test (smallest *p* value) among the items for which tests of equality of item difficulty parameters over time are significant.

For this item *j*, we then determine whether recalibration is the same in both groups (common recalibration) or not (differential recalibration) (Figure 2, step 3C). For this purpose, we test if the difference in item difficulty parameters over time is equal in the two groups with a Wald test: $H_{0}:\delta_{j\,p\,0}^{\left(2\right)}-\delta_{j\,p\,0}^{\left(1\right)}=\delta_{j\,p\,1}^{\left(2\right)}-\delta_{j\,p\,1}^{\left(1\right)}\,\forall p$

• If the test is significant at 5% level of significance, the null hypothesis of common recalibration in both groups is rejected and differential recalibration is assumed and will be tested within each group as follows.

• For each group *g* (*g* = 0, 1) the absence of recalibration for item *j* is tested: $H_{0}:\delta_{j\,p\,g}^{\left(1\right)}=\delta_{j\,p\,g}^{\left(2\right)}\,\forall p$ (Figure 2, step 3G) with a Bonferroni correction: $\alpha =\frac{5\%}{\mathrm{Number}\,\mathrm{of}\;\mathrm{groups}}$.

∘ If this test is not significant, we assume no recalibration and the model will be then updated with the following equality constraint for item *j* in group *g*: $\delta_{j\,p\,g}^{\left(1\right)}=\delta_{j\,p\,g}^{\left(2\right)}\,\forall p$.

∘ If this test is significant, we assume recalibration on item *j* in group *g* and we assess whether this recalibration is uniform (i.e., the differences in item difficulty parameters are the same for all response categories, Figure 2, step 3GU) with the following Wald test: $H_{0}:\delta_{j\,p\,g}^{\left(2\right)}-\;\delta_{j\,p\,g}^{\left(1\right)}=\Delta_{j\,g}\,\forall p$. If this test is significant, we assume non- uniform recalibration for item *j* in group *g*. In that case, when updating Model 3, the difficulty parameters $\delta_{j\,p\,g}^{\left(t\right)}$ will be freely estimated. If this test is not significant, we assume uniform recalibration and the model 3 will be then updated with the following constraints for item *j* in group *g*: $\delta_{j\,p\,g}^{\left(2\right)}-\delta_{j\,p\,g}^{\left(1\right)}=\Delta_{j\,g}\,\forall p$.

• If the test: $H_{0}:\delta_{j\,p\,0}^{\left(2\right)}-\delta_{j\,p\,0}^{\left(1\right)}=\delta_{j\,p\,1}^{\left(2\right)}-\delta_{j\,p\,1}^{\left(1\right)}\,\forall p$ is not significant (Figure 2, step 3C), common recalibration is assumed.

• Model 3 is updated appropriately by adding constraint of equal differences over time for both groups: $\delta_{j\,p\,0}^{\left(2\right)}-\delta_{j\,p\,0}^{\left(1\right)}=\delta_{j\,p\,1}^{\left(2\right)}-\delta_{j\,p\,1}^{\left(1\right)}\,\forall p$. The parameters of this updated model are estimated and we test whether this common recalibration can be considered uniform: $H_{0}:\delta_{j\,p\,g}^{\left(2\right)}-\delta_{j\,p\,g}^{\left(1\right)}=\Delta_{j}\,\forall p,g$(Figure 2, step 3CU).

∘ If this test is significant at 5% level of significance, we assume common non-uniform recalibration on item *j* which will be constrained as follows: $\delta_{j\,p\,0}^{\left(2\right)}-\delta_{j\,p\,0}^{\left(1\right)}=\delta_{j\,p\,1}^{\left(2\right)}-\;\delta_{j\,p\,1}^{\left(1\right)}\,\forall p$.

∘ If this test is not significant, we assume common uniform recalibration on item *j* which will be constrained as follows: $\delta_{j\,p\,0}^{\left(2\right)}-\delta_{j\,p\,0}^{\left(1\right)}=\delta_{j\,p\,1}^{\left(2\right)}-\delta_{j\,p\,1}^{\left(1\right)}\,\forall p$ and $\delta_{j\,p\,g}^{\left(2\right)}-\delta_{j\,p\,g}^{\left(1\right)}=\;\Delta_{j}\forall p,g$.

Model 3 is updated for item *j* according to the previous tests and associated constraints are added as appropriate (common or differential recalibration between groups, (non-)uniform recalibration within each group) during this first loop. Step 3 is repeated on the remaining items. The iterative process is stopped when no more differences on item difficulty parameters are detected or when differences are detected on *J*−1 items among *J* items, and we subsequently proceed to the last step (step 4).

#### Step 4: Estimation of the Covariate Effect on Latent Trait Change (Model 4)

The last step of the algorithm estimates the effect of the covariate g on the latent trait change adjusted on the differences in item difficulty parameters that were previously evidenced. Tests of group effect (*H*_0_:β = 0), time effect ($H_{0}:\mu_{0}^{\left(2\right)}=0$), and interaction effect (*H*_0_:β_inter_ = 0) are performed.

**FIGURE 3:**
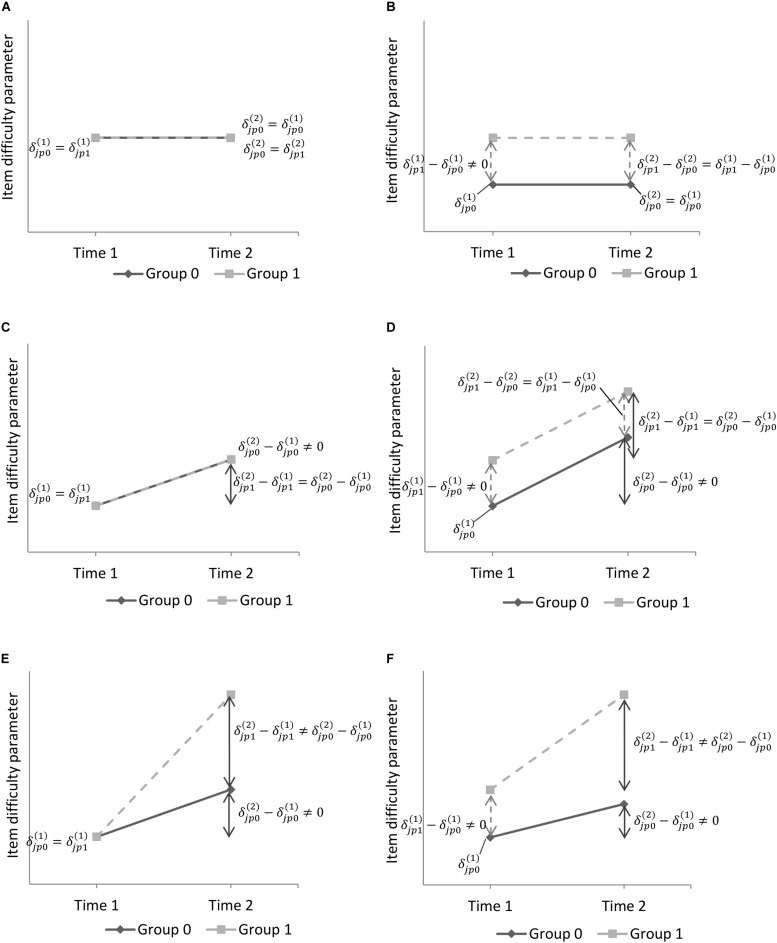
Illustrative graphs of item difficulty parameters change over time and between two groups. $\delta_{j\,p\,g}^{\left(t\right)}$ is the difficulty parameter of response category *p* of item *j* for group *g* at time *t*. **(A)** Same item difficulties between groups at *t* = 1, no recalibration. **(B)** Different item difficulties between groups at *t* = 1, no recalibration. **(C)** Same item difficulties at *t* = 1 and common recalibration between groups. **(D)** Different item difficulties at *t* = 1 and common recalibration between groups. **(E)** Same item difficulties at *t* = 1 and differential recalibration between groups. **(F)** Different item difficulties at *t* = 1 and differential recalibration between groups.

The effect of the group is also estimated on item functioning at time 1 (different item difficulty parameters between groups at the first measurement occasion) as well as on recalibration occurrence (common or differential recalibration, uniform or non-uniform recalibration). The different combinations of item difficulty parameters change over time and between two groups that can be detected appear in Figure 3. Values of an item difficulty parameter of an answer category p of an item j at each time and in each group are represented in each subfigure to draw the different possible changes.

## Illustrative Example–Application of Rosali to the ELCCA Study

We applied ROSALI on a longitudinal study named ELCCA that took place in the Department of Onco-dermatology at Nantes University Hospital and at Nantes-Angers Cancerology Institute in France. The aim of the ELCCA study was to compare HRQoL change over time between melanoma and breast cancer patients with early stage non-metastatic (stages I and II) cancer (Bourdon et al., 2016). Investigating whether changes in HRQoL and in patients’ adaptation (using recalibration RS analyses) differ according to cancer site can help identifying specific needs in terms of supportive care interventions during disease course.

During the first year following diagnosis, 293 patients (215 breast cancer and 78 melanoma) were followed and completed self-report questionnaires among which the cancer-specific Quality of Life Questionnaire (QLQ-C30 version 3.0) developed by the European Organization for Research and Treatment of Cancer (EORTC) (Aaronson et al., 1993). The QLQ-C30 is composed of five functional scales, nine symptom scales and a global health status scale. Among the domains assessed by the QLQ-C30, we focused on the emotional functioning scale as we assumed that aspects of quality of life related to mental health were likely to be affected by response shift. Emotional functioning was the latent trait of interest and was assessed within 1 month of diagnosis (time 1) and at the end of treatments i.e., 12 months later (time 2) using four items [(1) *Did you feel tense?*, (2) *Did you worry?*, (3) *Did you feel irritable?* and (4) *Did you feel depressed?*] with a four-point rating scale (response categories: *0-Very much*, *1-Quite a bit*, *2-A little*, *3-Not at all*). A high level of the latent trait represents high emotional functioning.

RespOnse Shift ALgorithm at Item-level was applied on this dataset to identify whether item functioning close to diagnosis, RS and emotional functioning change over time differ depending on cancer site. The output of the Stata module ROSALI applied on ELCCA data can be found in the Supplementary file.

### Detection of Differences in Item Difficulty Parameters Between Groups at Time 1 (Steps A–C)

Responses to the four items of the emotional functioning scale were modeled using a cross-sectional PCM at time 1 (model A) assuming different item difficulty parameters between breast cancer and melanoma patients. Model A was compared to model B assuming equal item functioning for both types of cancer at time 1. The test of no overall differences was significant (LRT, *p* = 0.029) and we subsequently proceeded to iterative step C to detect which items showed significantly different item difficulty parameters between groups.

Significant differences were found for item 3 “*Did you feel irritable?*” at the first iteration (chi-square statistic = 13.80, three degrees of freedom (df), *p* < 0.0125 with Bonferroni-adjusted significance level of 0.05/4). The hypothesis of uniform difference was not rejected (chi-square statistic = 4.27, 2 df, *p* > 0.05). Item difficulty parameters for item 3 were significantly lower in breast cancer patients at time 1 than in melanoma patients (difference in item difficulty parameters = −0.44, standard error (s.e.) = 0.22). It means that for a same level of emotional functioning, breast cancer patients tended to report lower levels of irritability in the month following diagnosis than melanoma patients.

The model was updated to take into account these differences on item 3 (uniform difference in item difficulty parameters at time 1 for breast cancer and melanoma patients). The second iteration of step C did not show difference in item difficulty parameters between groups for any of the three remaining items (item difficulty parameters common to breast cancer and melanoma patients for items 1, 2, and 4 at time 1).

### Recalibration Detection Between Time 1 and Time 2 (Steps 1–4)

A longitudinal PCM between time 1 and time 2 (model 1) with uniform differences in item difficulty parameters between groups at time 1 for item 3 was then fitted. In this model, differential recalibration over time, depending on the type of cancer, was estimated for all items. Model 1 was compared to Model 2 assuming no recalibration. The test of overall recalibration was significant (LRT, *p* < 0.001) and we subsequently proceeded to iterative step 3 to detect which items showed significant change in difficulty parameters over time.

At the first iteration of step 3, recalibration was found for item 3 (*Did you feel irritable?*) (chi-square statistic = 38.05, 6 df, *p* < 0.0125, Bonferroni-adjusted significance level of 0.05/4). The hypothesis of common recalibration between groups was not rejected (chi-square statistic = 6.01, 3 df, *p* > 0.05), thus the model was updated with common change in item difficulty parameters over time for both groups. The recalibration was assumed uniform, as the null hypothesis of common uniform recalibration in both groups was not rejected (chi-square statistic = 5.87, 2 df, *p* > 0.05). For both groups, all item difficulty parameters for item 3 were higher at time 2 than at time 1 (difference in item difficulties = 0.70, s.e. = 0.21). This change in item difficulty parameters corresponds to the Figure 3D. The solid line represents the change between the month following diagnosis and the end of treatments for breast cancer patients. The dashed line represents the change over time for melanoma patients. The common uniform recalibration for item 3 means that considering a same level of emotional functioning over time, melanoma and breast cancer patients tended to report higher levels of irritability at the end of treatments than in the month following diagnosis.

The second iteration of step 3 was performed on an updated longitudinal PCM that also took into account common uniform recalibration for item 3. Recalibration was detected on item 2 (*Did you worry?*, chi-square statistic = 23.06, 6 df, *p* < 0.0167, Bonferroni-adjusted significance level = 0.05/3). The hypothesis of common recalibration between groups was not rejected (chi-square statistic = 2.05, 3 df., *p* > 0.05), thus the model was simplified with common item difficulty parameters on item 2 at time 2 for both groups. The hypothesis of uniform recalibration was not rejected (chi-square statistic = 1.08, 2 df, *p* > 0.05). Item difficulty parameters for item 2 were lower at time 2 than at time 1 for both groups (difference in item difficulties = −0.96, s.e. = 0.21) (Figure 3C with decreasing item difficulty parameters). Hence, for a same level of emotional functioning, melanoma and breast cancer patients tended to report lower levels of worry 1 year after diagnosis than in the month following diagnosis.

The third iteration was performed on a model updated to include uniform recalibration common to both groups for item 2. This iteration did not show recalibration for the remaining two items.

In step 4, emotional functioning mean change over time was tested using a longitudinal PCM that took into account different item difficulty parameters between groups at time 1 for item 3 and uniform recalibration for items 2 and 3, common to both groups. Breast cancer patients showed significantly lower emotional functioning mean level in the month following the diagnosis than melanoma patients (estimated means in Table 1, type-of-cancer effect on the latent trait, *p* = 0.004). The emotional functioning level remained stable for melanoma patients during the first year following diagnosis (time effect on the latent trait, *p* = 0.78), whereas it significantly increased for breast cancer patients (type-of-cancer^∗^time interaction effect on the latent trait, *p* = 0.011). Final parameter estimates are presented in Table 1.

**TABLE 1:** Item parameter estimates of the final model for ROSALI (model 4) applied to the emotional functioning domain of ELCCA data at time 1 (within the month following diagnosis) and Time 2 (month 12).

Item	Response category	Time 1 1 month (post-diagnosis)		Time 2 (12 months post-diagnosis)	
		Melanoma	Breast cancer	Melanoma	Breast cancer
		$\delta_{j\,p\,0}^{\left(1\right)}$ (s.e)	$\delta_{j\,p\,1}^{\left(1\right)}$ (s.e)	$\delta_{j\,p\,0}^{\left(1\right)}$ (s.e)	$\delta_{j\,p\,1}^{\left(1\right)}$ (s.e)
Did you feel tense?					
	1	−4.53 (0.42)	$\delta_{j\,p\,0}^{\left(1\right)}$+0	$\delta_{j\,p\,0}^{\left(1\right)}$+0	$\delta_{j\,p\,0}^{\left(1\right)}$+0+0
	2	−2.70 (0.33)	$\delta_{j\,p\,0}^{\left(1\right)}$+0	$\delta_{j\,p\,0}^{\left(1\right)}$+0	$\delta_{j\,p\,0}^{\left(1\right)}$+0+0
	3	0.97 (0.31)	$\delta_{j\,p\,0}^{\left(1\right)}$+0	$\delta_{j\,p\,0}^{\left(1\right)}$+0	$\delta_{j\,p\,0}^{\left(1\right)}$+0+0
Did you worry?^a^					
	1	−3.36 (0.37)	$\delta_{j\,p\,0}^{\left(1\right)}$+0	$\delta_{j\,p\,0}^{\left(1\right)}$−0.96*(0.21)	$\delta_{j\,p\,0}^{\left(1\right)}$−0.96*+0
	2	−1.69 (0.32)	$\delta_{j\,p\,0}^{\left(1\right)}$+0	$\delta_{j\,p\,0}^{\left(1\right)}$−0.96*(0.21)	$\delta_{j\,p\,0}^{\left(1\right)}$−0.96*+0
	3	2.30 (0.33)	$\delta_{j\,p\,0}^{\left(1\right)}$+0	$\delta_{j\,p\,0}^{\left(1\right)}$−0.96*(0.21)	$\delta_{j\,p\,0}^{\left(1\right)}$−0.96*+0
Did you feel irritable?^b^					
	1	−4.94 (0.48)	$\delta_{j\,p\,0}^{\left(1\right)}$−**0.44^§^** (0.22)	$\delta_{j\,p\,0}^{\left(1\right)}$+0.70*(0.21)	$\delta_{j\,p\,0}^{\left(1\right)}$−0.44^§^ +0.70*
	2	−3.27 (0.37)	$\delta_{j\,p\,0}^{\left(1\right)}$−**0.44^§^** (0.22)	$\delta_{j\,p\,0}^{\left(1\right)}$+0.70*(0.21)	$\delta_{j\,p\,0}^{\left(1\right)}$−0.44^§^ +0.70*
	3	−0.12 (0.34)	$\delta_{j\,p\,0}^{\left(1\right)}$−**0.44^§^** (0.22)	$\delta_{j\,p\,0}^{\left(1\right)}$+0.70*(0.21)	$\delta_{j\,p\,0}^{\left(1\right)}$−0.44^§^ +0.70*
Did you feel depressed?					
	1	−4.75 (0.45)	$\delta_{j\,p\,0}^{\left(1\right)}$+0	$\delta_{j\,p\,0}^{\left(1\right)}$+0	$\delta_{j\,p\,0}^{\left(1\right)}$+0+0
	2	−3.18 (0.34)	$\delta_{j\,p\,0}^{\left(1\right)}$+0	$\delta_{j\,p\,0}^{\left(1\right)}$+0	$\delta_{j\,p\,0}^{\left(1\right)}$+0+0
	3	−0.25 (0.31)	$\delta_{j\,p\,0}^{\left(1\right)}$+0	$\delta_{j\,p\,0}^{\left(1\right)}$+0	$\delta_{j\,p\,0}^{\left(1\right)}$+0+0
Mean of latent trait : est. (s.e.)		0	−**0.95^§^** (0.33)	0.09 (0.33)	0.09*(0.35)
Variance of latent trait : est. (s.e.)		4.76 (0.65)	4.76 (0.65)	5.74 (0.80)	5.74 (0.80)

$\delta_{j\,p\,g}^{\left(1\right)}$: item difficulty parameter for each response category *p* > 0 of item *j* in group *g* at time *t*. **^§^** Parameter estimate significantly different between melanoma group and breast cancer group at time 1. *Parameter estimate significantly different between time 2 and time 1 within a given group. ^*a*^Uniform recalibration common to both groups. ^*b*^Different item difficulty parameters between groups at time 1. Uniform recalibration common to both groups. Est.: estimation, s.e.: standard error. Sizes of the significant effects between groups or times are in bold.

## Discussion

The RespOnse Shift ALgorithm at Item-level (ROSALI) was deeply modified to allow estimating the effects of a group covariate on item functioning, RS occurrence (uniform and non-uniform recalibration) and latent trait change. ROSALI thus enables the exploration of longitudinal measurement non-invariance with the estimation of latent trait change while accounting for RS but also considering RS as an outcome of interest and investigating whether RS is common or not between subgroups defined by a covariate. It is therefore possible to overcome the strong hypothesis that is usually made regarding similar patients’ adaptation where an average RS effect is estimated for all patients regardless of their characteristics. As an illustration, the application of ROSALI for assessing emotional functioning changes and RS in breast cancer and melanoma patients showed that melanoma patients tended to report higher levels of irritability in the month following diagnosis than breast cancer patients for a comparable level of emotional functioning. This could possibly reflect a different impact of the diagnosis in melanoma patients with more feelings of irritability, anger and possibly self-blame regarding illness as reported in the literature (Tesio et al., 2017). At the end of treatments however, both breast cancer and melanoma patients were more likely to report higher levels of irritability but also lower levels of worry as compared to the month following diagnosis, for a same level of emotional functioning. This shift in patients’ perception of the items may reflect the impact of cancer treatments often associated with fatigue and increased irritability (Gerber, 2017; Palesh et al., 2018) but also a potential adaptation process to illness with less apprehension regarding the perspective of the illness course. Accounting for different perceptions of irritability levels between cancer sites at 1 month following diagnosis and common recalibration thereafter, emotional functioning significantly increased during the first year following diagnosis for breast cancer patients, whereas it remained stable for melanoma patients.

Integrating covariates in IRT or RMT models is commonly used to investigate DIF under the perspective of measurement bias to detect and account for non-invariant items in cross-sectional group comparisons (Hardouin et al., 2012; Dunya et al., 2018; Oude Voshaar et al., 2019). Although longitudinal IRT models have been used to assess latent trait change over time and lack of invariance (Meade et al., 2005; Lee and Cho, 2017; Yau et al., 2018), the effects of covariates on latent trait change and shift in item parameters is seldom reported and cannot easily be performed in comprehensive statistical software. ROSALI can now be used to detect RS which can vary in occurrence and magnitude depending on a covariate of interest. A Stata module performing ROSALI with or without a group covariate is available on Boston College’s Statistical Software Components archive (Blanchin and Brisson, 2020).

We have favored the use of longitudinal PCM from Rasch family models as ROSALI based on RMT was shown to outperform ROSALI based on IRT in terms of recalibration RS detection without covariates (Blanchin et al., 2020). Consequently, only uniform and non-uniform recalibration can be detected and accounted for. Recalibration refers to a change in the respondent’s internal standards of measurement. Other types of RS exist, like reprioritization which corresponds to a change in the respondent’s values, that is to say a shift in the relative importance of items constituting the target construct (Schwartz and Sprangers, 1999). Reprioritization occurrence can be assessed using more flexible models, for instance generalized partial credit models (GPCM) from longitudinal IRT models, which estimate the discrimination parameter of the items. From a conceptual point of view, while the concept of reprioritization can make sense at domain level (e.g., emotional domain becoming more indicative of the latent construct over time as compared to the physical domain), one may wonder whether it does at item level. Indeed, this would mean that some items are becoming more or less indicative of the latent trait over time. This could reveal a multidimensionality issue rather than RS *per se* or also question the occurrence of reconceptualization implying a change in the definition of the latent construct itself. The effect of multidimensionality on longitudinal non-invariance of a set of calibrated items (i.e., item parameter drift) has been assessed in a simulation study in educational testing (Li, 2008). The discrimination parameters have been shown to be less invariant over time than the item difficulties in case of violation of the unidimensionality assumption of IRT. Hence, reprioritization at item-level operationalized as a change in discrimination parameters could be indicative of violation of unidimensionality.

In cross-sectional studies, DIF can be defined as a different perception of the measured PRO depending on the group membership at a specific time point. Here, as measurement invariance is investigated across groups and time, DIF can be defined as differences in item difficulties between groups detected at the first measurement occasion that remain stable over time. Hence, if differences in item difficulties across groups have been detected on an item at time 1 in the first part of ROSALI and if no recalibration (Figure 3B) or common recalibration (Figure 3D) has also been detected on this item, then it is possible to conclude with ROSALI that DIF occurs on this item.

Anchor methods are commonly used in DIF detection to place the estimated parameters on a common scale (Kopf et al., 2015). These anchor items are identified *a priori* and are assumed to be invariant during the DIF detection in which other item parameters are compared. ROSALI does not rely on a set of *a priori* anchor items that would be invariant between groups or across time. In fact, little prior knowledge regarding items that could be good anchor candidates is usually available. Furthermore, parameter estimates can be inaccurate and the false discovery rate of DIF might increase if some non-invariant items are included in the anchor set depending on the selection strategy of the anchor items and the number of non-invariant items (Wang, 2004; Woods, 2009). Hence, model A and model 1 of ROSALI are fully non-invariant regarding items. In the iterative steps for detection of different item difficulty parameters between groups (step C) and over time (recalibration, step 3), a backward approach was adopted starting with a fully invariant model that is improved step-by-step to lead to a parsimonious model that better fits the data. Given the full invariance of model B and model 2 and that all items stay invariant in step C and step 3 except items identified with difference across groups (step C) and items with RS (step 3), the approach in step C and step 3 is similar to the all-other anchor method in DIF detection. The all-other anchor method (Cohen et al., 1996) assumes that all items except the one studied are anchors avoiding *a priori* anchor selection.

Multiple testing in step C and step 3 increases the type I error α and may lead to over-detection of different item difficulty parameters between groups or over time. To overcome this issue, an adjustment of statistical significance for the number of tests performed in these steps through a Bonferroni correction was applied.

RespOnse Shift ALgorithm at Item-level integrating a covariate has been applied here on a clinical study and seems valuable but its performance needs to be evaluated. The performance of the first version of ROSALI based on RMT without a covariate, assessed in a simulation study (Blanchin et al., 2020), was satisfying in terms of recalibration detection, identification of items affected by recalibration, and type of recalibration. These promising results need to be confirmed with a simulation study on ROSALI integrating a covariate.

RespOnse Shift ALgorithm at Item-level in its current form only allows for recalibration detection between two measurement occasions, integrating a group covariate, and future developments are needed to simultaneously investigate the effects of more than one covariate reflecting different patients’ characteristics. For instance, taking into account individual clinical and psychological characteristics may give more insight into patients’ adaptation and adjustment to illness among these subgroups. Three different sets of covariates could be considered: a set affecting item functioning at the first time of measurement, a set affecting RS occurrence and a set affecting latent trait change. The sets of covariates could partly or entirely overlap, allowing different or identical covariates to be associated with item functioning, RS and latent trait changes.

To date, RS analyses are mainly carried out using two measurement occasions that are either previously identified (e.g., before and after a treatment) (Wu, 2016) or estimated and deduced from observed data (Salmon et al., 2017). Focusing on only two measurement occasions may be constrained by the experimental design and/or be of interest if one wishes to study the potential effect of a clearly identified event for all patients (e.g., diagnosis, initiation of treatment) on their experience (e.g., HRQoL) and their adaptation or maladaptation to the event. However, in a longitudinal study where there is not necessarily a clearly identified event that occurs at the same time for all patients, the choice and restriction to two measurement occasions is difficult to justify *a priori* and can be very restrictive. It is indeed very likely that RS does not occur in the same way and at the same time in all patients. Longitudinal Rasch models would have to be adapted to jointly take into account the latent trait and item parameters trajectories over time. Second-order latent growth models characterizing the relationships between the items and their underlying latent variable at each time point as well as the latent variable’s growth trajectory in one single specification (Proust-Lima et al., 2013; Isiordia and Ferrer, 2018) as well as Bayesian IRT models (Verhagen and Fox, 2013) could be an important lead to follow.

In conclusion, when analyzing the change of PRO over time, the use of ROSALI integrating a covariate can help exploring different patterns of item functioning, RS and latent trait changes between groups to better understand the way patients may experience adaptation to their illness depending on individual characteristics.

## Data Availability Statement

The data analyzed in this study is subject to the following licenses/restrictions: the SPHERE U1246 unit has signed a confidentiality agreement with the Institute of Cancer Research (ICO). Requests to access these datasets should be directed to Institut de cancérologie de l’Ouest, drci@ico.unicancer.fr.

## Ethics Statement

The studies involving human participants were reviewed and approved by Comité de Protection des Personnes hosted by Nantes University Hospital. The patients/participants provided their written informed consent to participate in this study.

## Author Contributions

KH performed the statistical analysis and wrote an initial draft. PB and MB developed the Stata module. All authors contributed to manuscript revision, read, and approved the submitted version.

## Conflict of Interest

The authors declare that the research was conducted in the absence of any commercial or financial relationships that could be construed as a potential conflict of interest.

## Supplementary Material

Supplementary File Output of the Stata module ROSALI applied on ELCCA data.
