# Supplementary material for: How to Investigate the Effects of Groups on Changes in Longitudinal Patient-Reported Outcomes and Response Shift Using Rasch Models
Source: Front Psychol. 2020 Dec 23;11:613482. doi: 10.3389/fpsyg.2020.613482 (PMC7786435; doi:10.3389/fpsyg.2020.613482)
Supplement: Supplementary File — Output of the Stata module ROSALI applied on ELCCA data. [file Data_Sheet_1.PDF]

Syntax

```
*Install ROSALI package from SSC
ssc install rosali

*open dataset
use "rosali_example.dta",clear
*data must be presented in wide format: 1 line by individual

/*************************************************************************
* VARIABLE NAMES
* M1tense M1worry M1irrit M1depr: item responses at 1-month post diagnosis
* M12tense M12worry M12irrit M12depr: item responses at 12-month post diagnosis
* cancer: type of cancer
*************************************************************************/
/*************************************************************************
* VARIABLE CODING
* M1tense-M12depr: 0 "very much", 1 "quite a bit", 2 "a little", 3 "not at all"
* cancer: 0 "melanoma", 1 "breast cancer"
*************************************************************************/

*Perform ROSALI with a covariate (group option)
*Display all estimated models (detail option)
rosali M1tense-M1depr M12tense-M12depr,detail group(cancer)
```

Extract from the dataset

File Edit View Data Tools

idpat[1] 1

	M1tense	M1worry	M1irrit	M1depr	M12tense	M12worry	M12irrit	M12depr	cancer
38	2	0	2	0	1	1	1	1	1
39	.	.	.	.	2	2	3	1	1
40	2	2	2	2	2	2	2	2	1
41	2	2	2	3	3	2	3	3	1
42	0	0	1	1	1	0	0	1	1
43	3	3	3	3	3	3	2	2	1
44	2	2	3	3	3	3	3	3	1
45	2	2	3	2	3	3	3	2	1
46	3	3	3	3	3	3	3	3	1
47	0	.	1	2	2	1	1	2	1
48	0	0	0	0	3	1	2	3	1
49	2	2	3	3	3	3	3	3	1
50	2	2	3	3	2	2	2	2	1
51	3	3	3	3	3	3	3	3	1
52	2	2	3	3	3	3	3	3	1
53	2	2	3	2	2	2	2	3	1
54	3	3	3	3	3	3	3	3	1
55	2	2	3	3	.	.	3	3	1
56	2	0	2	2	3	3	3	3	1

Output

```
. rosali M1tense-M12depr,detail group(cancer)

    ---------------------------------------------------------------------------
              Time 1                     Time 2                 Nb of Answer Cat.
    ---------------------------------------------------------------------------
              M1tense                    M12tense               4
              M1worry                    M12worry               4
              M1irrit                    M12irrit               4
              M1depr                     M12depr                4
    ---------------------------------------------------------------------------
         Nb of patients: cancer 0 = 78 ; cancer 1 = 215
    ---------------------------------------------------------------------------
```

For all models : - mean of the latent trait in cancer 0 at time 1 is constrained at 0
- equality of variances between groups

PART 1: DETECTION OF DIFFERENCE IN ITEM DIFFICULTIES BETWEEN GROUPS AT TIME 1

PROCESSING STEP A

MODEL A: Overall measurement non-invariance between groups

Item difficulties: estimates (s.e.)

	cancer=0	cancer=1
M1tense		
1	-5.56 (1.19)	-3.26 (0.42)
2	-2.33 (0.48)	-1.65 (0.27)
3	0.84 (0.41)	2.22 (0.30)
M1worry		
1	-3.38 (0.64)	-2.10 (0.33)
2	-1.30 (0.44)	-0.94 (0.26)
3	2.03 (0.47)	3.36 (0.37)
M1irrit		
1	-3.68 (0.88)	-4.35 (0.56)
2	-3.32 (0.58)	-2.30 (0.32)
3	0.30 (0.40)	-0.01 (0.25)
M1depr		
1	-4.56 (0.95)	-3.66 (0.49)
2	-2.57 (0.55)	-2.24 (0.31)
3	-0.84 (0.41)	0.62 (0.25)

Latent trait distribution

	Estimate	Standard error
Variance	4.39	0.69
Group effect	0 (constrained)	

No group effect: equality of the latent trait means between groups
All item difficulties are freely estimated in both groups

PROCESSING STEP B

MODEL B: Overall measurement invariance between groups

Item difficulties: estimates (s.e.)

	cancer=0	cancer=1
M1tense		
1	-4.21 (0.47)	-4.21 (0.47)
2	-2.41 (0.34)	-2.41 (0.34)
3	1.20 (0.32)	1.20 (0.32)
M1worry		
1	-2.97 (0.39)	-2.97 (0.39)
2	-1.62 (0.32)	-1.62 (0.32)
3	2.33 (0.36)	2.33 (0.36)
M1irrit		
1	-4.76 (0.55)	-4.76 (0.55)
2	-3.13 (0.37)	-3.13 (0.37)
3	-0.52 (0.31)	-0.52 (0.31)
M1depr		
1	-4.43 (0.51)	-4.43 (0.51)
2	-2.91 (0.37)	-2.91 (0.37)
3	-0.37 (0.31)	-0.37 (0.31)

Latent trait distribution

	Estimate	Standard error	P-value
Variance	4.22	0.67	
Group effect	-0.81	0.31	0.0098

Group effect estimated: mean of the latent trait of group 1 freely estimated
Equality of the item difficulties between groups

LIKELIHOOD-RATIO TEST

Model A vs Model B

Chi-square	DF	P-value
21.46	11	0.0289

DIFFERENCE IN ITEM DIFFICULTIES BETWEEN GROUPS LIKELY

PROCESSING STEP C

Loop 1
Adjusted alpha: 0.0125

Freed item	Chi-Square	DF	P-Value
M1tense	4.222	3	0.2385
M1worry	2.303	3	0.5119
M1irrit	13.795	3	0.0032
M1depr	3.769	3	0.2876

Difference between groups on M1irrit at time 1

Test of uniform difference

Chi-square	DF	P-value
4.27	2	0.12

M1irrit : Uniform differences of item difficulties between groups at T1

Loop 2
Adjusted alpha: 0.0167

Freed item	Chi-Square	DF	P-Value
M1tense	2.354	3	0.5022
M1worry	2.858	3	0.4140
M1depr	2.899	3	0.4074

No other significant tests

PART 2 : DETECTION OF DIFFERENCE IN ITEM DIFFICULTIES ACROSS TIME (RECALIBRATION)

PROCESSING STEP 1

MODEL 1: Overall longitudinal measurement non-invariance across time (RS on all items)

Item difficulties: estimates (s.e.)

	Time 1		Time 2	
	cancer=0	cancer=1	cancer=0	cancer=1
M1tense				
1	-4.47 (0.48)	-4.47 (0.48)	-4.08 (0.84)	-6.39 (0.71)
2	-2.62 (0.35)	-2.62 (0.35)	-3.02 (0.59)	-4.11 (0.45)
3	1.04 (0.33)	1.04 (0.33)	0.71 (0.47)	-0.21 (0.40)
M1worry				
1	-3.20 (0.40)	-3.20 (0.40)	-4.06 (0.84)	-6.50 (0.70)
2	-1.83 (0.33)	-1.83 (0.33)	-3.07 (0.59)	-3.69 (0.44)
3	2.20 (0.36)	2.20 (0.36)	1.43 (0.48)	0.24 (0.40)
M1irrit				
1	-4.29 (0.56)	-5.21 (0.57)	-4.31 (0.93)	-6.59 (0.76)
2	-2.66 (0.40)	-3.57 (0.40)	-3.60 (0.64)	-4.37 (0.47)

3	-0.04 (0.35)	-0.95 (0.34)	0.40 (0.46)	-0.64 (0.39)
M1depr				
1	-4.69 (0.52)	-4.69 (0.52)	-3.76 (0.89)	-6.92 (0.81)
2	-3.14 (0.38)	-3.14 (0.38)	-3.69 (0.67)	-4.48 (0.48)
3	-0.55 (0.32)	-0.55 (0.32)	-0.06 (0.46)	-1.14 (0.39)

Latent trait distribution

	Estimate	Standard error	P-value
Variance Time 1	4.36	0.69	
Variance Time 2	6.61	1.04	
Covariance	2.90	0.49	
Group effect	-1.03	0.33	0.0015
Time effect	0 (constrained)		
TimexGroup inter	0 (constrained)		

Group effect estimated: mean of the latent trait of group 1 at time 1 freely estimated

No time effect: equality of means of the latent trait of group 0 across time

All item difficulties freely estimated across time

PROCESSING STEP 2

MODEL 2: Overall longitudinal measurement invariance across time (no RS)

Item difficulties: estimates (s.e.)

	Time 1		Time 2	
	cancer=0	cancer=1	cancer=0	cancer=1
M1tense				
1	-4.33 (0.40)	-4.33 (0.40)	-4.33 (0.40)	-4.33 (0.40)
2	-2.58 (0.31)	-2.58 (0.31)	-2.58 (0.31)	-2.58 (0.31)
3	1.00 (0.29)	1.00 (0.29)	1.00 (0.29)	1.00 (0.29)
M1worry				
1	-3.49 (0.36)	-3.49 (0.36)	-3.49 (0.36)	-3.49 (0.36)
2	-2.00 (0.30)	-2.00 (0.30)	-2.00 (0.30)	-2.00 (0.30)
3	1.75 (0.30)	1.75 (0.30)	1.75 (0.30)	1.75 (0.30)
M1irrit				
1	-4.40 (0.45)	-4.85 (0.46)	-4.40 (0.45)	-4.85 (0.46)
2	-2.81 (0.34)	-3.26 (0.34)	-2.81 (0.34)	-3.26 (0.34)
3	0.28 (0.31)	-0.17 (0.30)	0.28 (0.31)	-0.17 (0.30)
M1depr				
1	-4.53 (0.43)	-4.53 (0.43)	-4.53 (0.43)	-4.53 (0.43)
2	-3.04 (0.33)	-3.04 (0.33)	-3.04 (0.33)	-3.04 (0.33)
3	-0.20 (0.29)	-0.20 (0.29)	-0.20 (0.29)	-0.20 (0.29)

Latent trait distribution

	Estimate	Standard error	P-value
Variance Time 1	4.24	0.58	
Variance Time 2	5.55	0.77	
Covariance	2.63	0.45	
Group effect	-0.91	0.31	0.0037
Time effect	0.20	0.31	0.5189
TimexGroup inter	0.92	0.36	0.0110

Group effect estimated: mean of the latent trait of group 1 at time 1 freely estimated

Time effect estimated: mean of the latent trait of group 0 at time 2 freely estimated

Equality of all item difficulties across time

LIKELIHOOD-RATIO TEST

Model 1 vs Model 2

Chi-square	DF	P-value
76.00	22	0.0000

DIFFERENCE IN ITEM DIFFICULTIES ACROSS TIME LIKELY

PROCESSING STEP 3

Loop 1
    Adjusted alpha : 0.0125

Freed item	Chi-Square	DF	P-Value
M1tense	2.535	6	0.8646
M1worry	36.572	6	0.0000
M1irrit	38.046	6	0.0000
M1depr	9.234	6	0.1608

Recalibration on M1irrit

Test	Chi-Square	DF	P-Value
Common RC?	6.01	3	0.1113
Uniform RC?	5.87	2	0.0530

M1irrit : Uniform common RC

Loop 2
    Adjusted alpha : 0.0167

Freed item	Chi-Square	DF	P-Value
M1tense	2.363	6	0.8835
M1worry	23.064	6	0.0008
M1depr	20.902	6	0.0019

Recalibration on M1worry

Test	Chi-Square	DF	P-Value
Common RC?	2.05	3	0.5628
Uniform RC?	1.08	2	0.5840

M1worry : Uniform common RC

Loop 3
    Adjusted alpha : 0.0250

Freed item	Chi-Square	DF	P-Value
M1tense	5.760	6	0.4506
M1depr	8.549	6	0.2006

No other significant tests

SUMMARY

Item	Difference in groups at T1	Recalibration	RC cancer 0	RC cancer 1
M1tense				
M1worry		Common	Uniform	Uniform
M1irrit	Uniform	Common	Uniform	Uniform
M1depr				

PROCESSING STEP 4

    MODEL 4 = Final model

Item difficulties: estimates (s.e.)

	Time 1		Time 2	
	cancer=0	cancer=1	cancer=0	cancer=1
M1tense				
1	-4.53 (0.42)	-4.53 (0.42)	-4.53 (0.42)	-4.53 (0.42)
2	-2.70 (0.33)	-2.70 (0.33)	-2.70 (0.33)	-2.70 (0.33)
3	0.97 (0.31)	0.97 (0.31)	0.97 (0.31)	0.97 (0.31)
M1worry				
1	-3.36 (0.37)	-3.36 (0.37)	-4.32 (0.42)	-4.32 (0.42)
2	-1.69 (0.32)	-1.69 (0.32)	-2.65 (0.36)	-2.65 (0.36)
3	2.30 (0.33)	2.30 (0.33)	1.34 (0.34)	1.34 (0.34)
M1irrit				
1	-4.94 (0.48)	-5.38 (0.48)	-4.23 (0.48)	-4.68 (0.49)
2	-3.27 (0.37)	-3.71 (0.36)	-2.56 (0.38)	-3.00 (0.38)
3	-0.12 (0.34)	-0.57 (0.32)	0.58 (0.36)	0.14 (0.35)
M1depr				
1	-4.75 (0.45)	-4.75 (0.45)	-4.75 (0.45)	-4.75 (0.45)
2	-3.18 (0.34)	-3.18 (0.34)	-3.18 (0.34)	-3.18 (0.34)
3	-0.25 (0.31)	-0.25 (0.31)	-0.25 (0.31)	-0.25 (0.31)

Latent trait distribution

	Estimate	Standard error	P-value
Variance Time 1	4.76	0.65	
Variance Time 2	5.74	0.80	
Covariance	2.82	0.48	
Group effect	-0.95	0.33	0.0041
Time effect	0.09	0.33	0.7815
TimexGroup inter	0.94	0.37	0.0109

Estimates of differences between groups and recalibration

	Difference of groups at T1	RECALIBRATION	
		cancer=0	cancer=1
M1tense			
1	0.00 (0.00)	0.00 (0.00)	0.00 (0.00)
2	0.00 (0.00)	0.00 (0.00)	0.00 (0.00)
3	0.00 (0.00)	0.00 (0.00)	0.00 (0.00)
M1worry			
1	0.00 (0.00)	-0.96 (0.21)	-0.96 (0.21)
2	0.00 (0.00)	-0.96 (0.21)	-0.96 (0.21)
3	0.00 (0.00)	-0.96 (0.21)	-0.96 (0.21)
M1irrit			
1	-0.44 (0.22)	0.70 (0.21)	0.70 (0.21)
2	-0.44 (0.22)	0.70 (0.21)	0.70 (0.21)
3	-0.44 (0.22)	0.70 (0.21)	0.70 (0.21)
M1depr			
1	0.00 (0.00)	0.00 (0.00)	0.00 (0.00)
2	0.00 (0.00)	0.00 (0.00)	0.00 (0.00)
3	0.00 (0.00)	0.00 (0.00)	0.00 (0.00)
